# Crowdsourcing genomic analyses of ash and ash dieback – power to the people

**DOI:** 10.1186/2047-217X-2-2

**Published:** 2013-02-12

**Authors:** Dan MacLean, Kentaro Yoshida, Anne Edwards, Lisa Crossman, Bernardo Clavijo, Matt Clark, David Swarbreck, Matthew Bashton, Patrick Chapman, Mark Gijzen, Mario Caccamo, Allan Downie, Sophien Kamoun, Diane GO Saunders

**Affiliations:** 1 The Sainsbury Laboratory, Norwich Research Park, Norwich NR4 7UH, UK; 2 The John Innes Centre, Norwich Research Park, Norwich, NR4 7UH, UK; 3 The Genome Analysis Centre, Norwich Research Park, Norwich, NR4 7UH, UK; 4 Bioinformatics Support Unit, Faculty of Medical Sciences, Framlington Place, Newcastle University, Newcastle, NE2 4HH, UK; 5 Agriculture and Agri-food Canada, 1391 Sandford St, London, ON, N5V 4T3, Canada

**Keywords:** Crowdsource, Genomics, Ash dieback, Open source, Altmetrics

## Abstract

Ash dieback is a devastating fungal disease of ash trees that has swept across Europe and recently reached the UK. This emergent pathogen has received little study in the past and its effect threatens to overwhelm the ash population. In response to this we have produced some initial genomics datasets and taken the unusual step of releasing them to the scientific community for analysis without first performing our own. In this manner we hope to ‘crowdsource’ analyses and bring the expertise of the community to bear on this problem as quickly as possible. Our data has been released through our website at oadb.tsl.ac.uk and a public GitHub repository.

## Main text

### oadb.tsl.ac.uk: A new resource for the crowdsourcing of genomic analyses on ash and ash dieback

Ash dieback is a devastating disease of ash trees caused by the aggressive fungal pathogen *Chalara fraxinea*. This fungus emerged in the early 1990s in Poland and has since spread west across Europe reaching native forests in the UK late last year. The emergence of *Chalara* in the UK caused public outcry where up to 90% of the more than 80 million ash trees are thought to be under threat. The disease, which is a newcomer to Britain, was first reported in the natural environment in October 2012 and has since been recorded in native woodland throughout the UK. There is no known treatment for ash dieback, current control measures include burning infected trees to try and prevent spread [1] and the implications for the UK environment and the economy remain stark.

To kick start genomic analyses of the pathogen and host, we took the unconventional step of rapidly generating and releasing genomic sequence data. We released the data through our new ash and ash dieback website, oadb.tsl.ac.uk, which we launched in December 2012. Speed is essential in responses to rapidly appearing and threatening diseases and with this initiative we aim to make it possible for experts from around the world to access the data and analyse it immediately, speeding up the process of discovery. We hope that by providing data as soon as possible we will stimulate crowdsourcing and open community engagement to tackle this devastating pathogen.

### The transcriptomics and genomics data we have released so far

We have generated and released Illumina sequence data of both the transcriptome and genome of *Chalara* and the transcriptome of infected and uninfected ash trees. We took the unusual first step of directly sequencing the “interaction transcriptome” [2] of a lesion dissected from an infected ash twig collected in the field. This enabled us to respond quickly, generating useful information without time-consuming standard laboratory culturing; the shortest route from the wood to the sequencer to the computer.

The *Chalara* transcriptome data, generated at The Sainsbury Laboratory (TSL, Norwich, UK) was derived from two infected ash samples collected at Ashwellthorpe Lower Wood, near Norwich; the location of the first confirmed case of ash dieback in the wild in the UK. Here we extracted RNA from branches of two infected ash trees, prepared cDNA libraries from each and sequenced these to create 76 nt paired-end reads on our Illumina GAII.

In parallel to the transcriptome data, genome sequence data were produced in a coordinated effort between The John Innes Centre (JIC), TSL and The Genome Analysis Centre (TGAC) in Norwich. A single *C. fraxinea* isolate was cultured from infected tissue found in Kenninghall Wood. Genomic DNA libraries were constructed and sequenced on an Illumina MiSeq sequencer as 150 nt and 250 nt paired-end libraries.

As soon as these datasets were generated we released them through oadb.tsl.ac.uk. We took the unusual step to release the data before preliminary analysis had been undertaken so that we might take advantage of the huge range of expertise and knowledge available outside our groups, and thereby make the best of the data as quickly as possible via a crowdsourcing approach.

### Crowdsourcing: bringing the power of many, marshalling expertise and democratising genomics

Crowdsourcing is a form of massively parallel collaboration, the main distinguishing feature of which is the low overhead to entry of participation and low level of investment from a participant. The power is in the sheer number of people interested in seeing the goals of the project achieved. Scientists have not been slow to adopt these models to carry out work that could not be automated successfully and require human intelligence and expertise. Recently genomic scientists have made inroads to leveraging the power of crowds to annotate and assemble the genome sequence of a novel strain of *Escherichia coli* O104:H4 bacteria that caused a serious outbreak of foodborne illness in northern Germany in spring 2011. These scientists were able to quickly link up with others across the world with similar skills to rapidly analyse the novel pathogenic strain [3]. Most importantly, crowdsourcing allows for a new form of potentially effective live peer-review, many sets of eyes interrogating and reviewing data and analyses mean that unusual results are quickly highlighted and can be assessed and dealt with appropriately. Whether they are eventually found to be inconsistencies in analysis or more exciting genuine new discoveries, the end product is brought to the scientific community many times faster than the usual peer-review by a small number of reviewers and crucially it all happens out in the open with maximum transparency. The cornerstone of our crowdsourcing is our repository on GitHub [4], a versioning system designed for collaboration in software development that automatically maintains attribution of contribution, meaning that whoever contributes will get full credit for the difference that they made.

We are certain that the data will prove useful to anyone who wishes to be involved in the fightback against ash dieback and that concerted, early data-sharing and open analysis is a crucial step in a productive and timely response to emergent pathogen threats.

### The future of our data and our initiative

To date, genome analysis of emerging plant pathogens is not rapidly implemented as is routinely done with human pathogens [5,6]. Worse, the data (when available) is not immediately released into the public domain. We hope our openness will encourage the scientific community to engage in this proactive and collaborative model of working when faced with pressing challenges. Already we are seeing a significant amount of work being provided by external groups. Contributions of transcriptome assemblies, protein domain annotations, phylogenetic trees and BLASTs for specific gene family members have been provided from groups across the world.

### Credit where credit is due

We absolutely understand the need for scientists to be credited for what they do and we intend to make sure that everyone who contributes receives full attribution. The GitHub repository ensures this, and we are committed to the principle for all other potential results from this initiative. The altmetrics movement is making it possible and acceptable for scientists to cite the varied products of science [7], rather than simply the papers they write and we intend to make it as easy as possible for contributors to be able to cite what they did via commit number and potentially DOIs.

### Towards a rapid response for food and ecosystem security

A pathogenic threat to our forests and ecosystems is a threat to our ability to live on the planet sustainably, just as a threat to our crops is a threat to our ability to feed ourselves. In these situations it is vital to respond as quickly as possible so we must embrace the evolution of a new digital immune system [8]. Our initiative is an early step towards developing the crucial function of the digital immune system for response to plant pathogens; the thing we cannot upload to a repository is the people with the expertise and the will to contribute, and that is why we need the scientific community to download our data and provide analyses.

Our website and repository can be found at:

http://oadb.tsl.ac.uk

https://github.com/ash-dieback-crowdsource/data

## Abbreviations

DOI: Digital Object Identifier; JIC: The John Innes Centre; Nt: Nucleotide; TGAC: The Genome Analysis Centre; TSL: The Sainsbury Laboratory.

## Competing interests

The authors declare that they have no competing interests.

## Authors’ contributions

All authors contributed to the drafting of the manuscript. DM created the oadb website, designed and instantiated the GitHub repository and wrote the commentary, AE and AD sourced biological materials for sequencing, DGOS, KY and SK prepared biological materials, managed sequencing and performed analyses and contributed data to the repository. LC, BC, DS, M Clarke, PC and MG and MC provided analyses of data in the repository. All authors read and approved the final manuscript.

## References

[B1] Forestry commission Great Britain, Chalara dieback of ashhttp://www.forestry.gov.uk/chalara

[B2] Birch PRJ Kamoun S Studying interaction transcriptomes: coordinated analyses of gene expression during plant-microorganism interactions New Technologies for Life Sciences: A Trends Guide 2000 New York: Elsevier Science 77 82

[B3] GitHub crowdsource BGI data https://github.com/ehec-outbreak-crowdsourced/BGI-data-analysis/wiki

[B4] GitHub ash dieback crowdsource datahttps://github.com/ash-dieback-crowdsource/data

[B5] Green S Studholme DJ Laue BE Dorati F Lovell H Arnold D Cottrell JE Bridgett S Blaxter M Huitema E Thwaites R Sharp PM Jackson RW Kamoun S Comparative genome analysis provides insights into the evolution and adaptation of Pseudomonas syringae pv. aesculi on Aesculus hippocastanum PLoS One 2010 5 e10224 10.1371/journal.pone.0010224 20419105 PMC2856684

[B6] Kamoun S Genomics of emerging plant pathogens: too little, too late Microbiol Today 2012 39 140

[B7] Piwowar H Altmetrics: Value all research products Nature 2013 493 159 23302843 10.1038/493159a

[B8] Schatz MC Phillippy AM The rise of a digital immune system GigaScience 2012 1 4 23587178 10.1186/2047-217X-1-4 PMC3617452

